# Prevalence and determinants of unintended childbirth in Ethiopia

**DOI:** 10.1186/1471-2393-14-326

**Published:** 2014-09-18

**Authors:** Yibeltal Tebekaw, Bezuhan Aemro, Charles Teller

**Affiliations:** World Health Organization, Juba, Republic of South Sudan; BZY Research Consult, Addis Ababa, Ethiopia; Department of Global Health, Milken Institute of Public Health, George Washington University, Washington, DC, USA

**Keywords:** Women, Unintended pregnancy, Determinants

## Abstract

**Background:**

Ethiopia’s population policy specifically aims to reduce TFR from 7.7 to 4.0 and to increase contraceptive use from 4.0% to 44.0% between 1990 and 2015. In 2011, the use of contraceptive methods increased seven-fold from 4.0% to 27%; and the TFR declined by 38% to 4.8. The use of modern contraceptives is, however, much higher in the capital Addis Ababa (56%) and other urban areas but very low in rural areas (23%) far below the national average (27%). In 2011, one in four Ethiopian women had an unmet need for contraception. The main aim of this study was to assess the pattern and examine the socioeconomic and demographic correlates of unintended childbirth among women 15-49 years in Ethiopia.

**Methods:**

Data from the 2011 nationally representative Ethiopia Demographic and Health Survey are used. It covered 16,515 women of which 7,759 had at least one birth and thus included for this study. Multivariate logistic regression is used to see the net effects of each explanatory variable over the outcome variable.

**Results:**

The study found that nearly one in three (32%) births was unintended; and about two-thirds of these were mistimed. The regression model shows that the burden of unintended births in Ethiopia falls more heavily on young, unmarried, higher wealth, high parity, and ethnic majority women and those with less than secondary education and with large household size. These variables showed statistical significance with the outcome variable.

**Conclusion:**

The study found a relatively high prevalence of unintended childbirth in Ethiopia and this implies high levels of unmet need for child spacing and limiting. There is much need for better targeted family planning programs and strategies to strengthen and improve access to contraceptive services, to raise educational levels, and related information and communication particularly for those affected groups including young, unmarried, multipara, and those with less than secondary level of education. Further quantitative and qualitative research on the consequences of unintended pregnancy and childbirth related to prenatal and perinatal outcomes are vital to document process of change in the problem overtime.

## Background

Globally an estimated 80 million unintended pregnancies, both mistimed and unwanted, occur each year [1]. Nearly one in every 10 of the pregnancies ends up with unsafe abortions [2]. About 13% of deaths in 2008 were due to unsafe abortion; 62% of them were from Africa [1], many from unintended pregnancies that were unsafely aborted [3]. Every year, nearly 6.2 million African (5.5 million from sub Sahara) women have an unsafe abortion; and 29,000 of them die from the procedure [1]. Unintended pregnancy and births have grave consequences to the mother and family and are global social and health burdens [1, 4, 5]. Unintended pregnancies mostly arise as a result of nonuse or incorrect use of contraceptives, or a noticeable contraceptive failure [4, 6]. In 2007, more than one in every five African women of childbearing age had unmet need for family planning. About five percent of the contraceptive users face unexpected pregnancy due to contraceptive failure [1].

In Ethiopia, hundreds die in health facilities each year from abortion-related complications, but many more suffer from injuries or illness related to unsafe procedures [7]. Abortion is legal in Ethiopia since 2005 in cases of rape, incest or fetal impairment. In addition, a woman can legally terminate a pregnancy if her life or her child’s life is in danger, or if continuing the pregnancy or giving birth endangers her life [8].

Ethiopia’s population policy was developed in 1993 just before the UN’s International Conference on Population and Development in 1994, which adopted the principle that every pregnancy should be planned and wanted [9, 10]. The National Population Policy specifically aims to reduce TFR from 7.7 to 4.0 and to increase contraceptive use from 4.0% to 44.0% between 1990 and 2015. According to the 2011 EDHS report, the use of contraceptive methods increased sevenfold in two decades from 4.0% to 27%; and the TFR declined by 38% from 7.7 to 4.8 between 1990 and 2011. Modern method use in rural areas was very low (23%) far below the national average. The level of modern contraceptive use in Ethiopia is encouraging news for improving the health of women and their families. However, it is impossible to meet women’s fertility or reproductive goals with existing large number of mistimed or unwanted pregnancies. The country is generally characterized by high fertility, low contraceptive use, high maternal mortality (673 deaths per 100,000 live births) and high unmet need for family planning services [11].

In Ethiopia, the percentage of births that were unwanted or unplanned at the time of conception was 37%, 35% and 28% in the 2000, 2005, and 2011 respectively [11]. A cross-sectional study from Eastern Ethiopia shows that 33.3% of sexually active women had their most recent pregnancies classified unintended and the prevalence of unintended childbirth was 14.4% [12]. Another study from the same region shows a 27.9% unintended pregnancy level [13]. A nationwide facility based study shows 42% unintended pregnancies [14]. The studies indicate that younger, unmarried, and multipara women and those among the poor wealth quintiles have higher experience of unintended pregnancies [13, 15].

Previous studies in Ethiopia focused on unintended pregnancies not on unintended childbirths and have relied largely on low-scale or localized facility and community-based surveys [13, 15]. Little is therefore known about the determinants of unintended childbirth from diverse socio-economic and demographic contexts at national level. The current study compares women who had unintended births with all other women with history of childbirth. It intends to help policymakers and program designers in Ethiopia understand the extent and correlates of unintended childbirths as such evidence is an essential part in improving the reproductive health service delivery to the needy. The main aim of this study was to assess the pattern and examine the socioeconomic and demographic correlates of unintended childbirth among women 15-49 years in Ethiopia.

## Methods

The data for this paper were drawn from the 2011 nationally representative Ethiopia Demographic and Health Survey (EDHS). This is a secondary analysis of data. Authorization was obtained from the ICF International to download data from the Demographic and Health surveys (DHS) on-line archive and analyze and present findings. The survey was implemented by the Ethiopian Central Statistical Agency (CSA) with the technical assistance of ICF International through the MEASURE DHS project. The survey enquires about household members’ and individual characteristics using Household Questionnaire, Woman’s Questionnaire and Man’s Questionnaire. Individual women of reproductive age (15-49 years) were interviewed face to face on their background characteristics as well as on fertility and family planning behaviour, child mortality, adult and maternal mortality, nutritional status of women and children, the utilization of maternal and child health services, knowledge of HIV/AIDS and prevalence of HIV/AIDS and anaemia [11].

The sample was weighted to make the survey base more accurately representative of the population from which the sample was taken. Thus the descriptive analyses for this paper were based on weighted figures. However, since the multivariate analyses preserve the one respondent-one-response relationship, data were not weighted. The present analysis is restricted to last born children in the five years preceding the survey. EDHS tries to assess the level of unwanted fertility among women age 15-49 through a series of survey questions asked about each of the children born to them in the preceding five years (including current pregnancy). Women were asked about their last birth whether they wanted it then, wanted later, or did not want to have any more children at all. The term “wanted” permits identifying those mistimed pregnancies or births that occurred sooner than desired. In this study, if the birth or pregnancy was wanted then, it was considered to be *intended*; if it was wanted but at a later time, it was considered to be *mistimed*, and if it was not wanted at the time of conception, it was considered to be *unwanted* [11]. The dependent variable of interest in this study is therefore measured as a two-outcome variable and coded as *intended* birth, if the last childbirth occurred at a time when the woman wanted it, and *unintended* birth, if the pregnancy or last childbirth occurred at a time when the woman would have wanted it later or did not want it at all. Hence, unintended birth is estimated as the proportion of births resulting from unintended pregnancies.

Both bivariate and multivariate analyses were done to determine the presence of statistically significant associations and strength of associations between explanatory variables and the dependent variable. For this study, p-value of 0.05 was considered as significant level. The multivariate models (adjusted odds ratio) included variables that were significantly associated with the dependent variable (p-value < 0.05) in the bivariate analyses or crude odds ratios. The Hosmer and Lemeshow goodness of fit test showed P-value of 0.89 and Nagelkerke R Square value was 0.63 for the final model which shows that our data fairly fits with the logistic regression model. Multi-collinearities were also checked among selected variables including age versus parity, educational status versus working status, and educational status versus wealth index. The Variance Inflation Factor (VIF) and adjusted R^2^ values for each of the pairs ranged from 1.01- 1.31 and 0.001-0.011 respectively. Commonly, a VIF of 10 and above or a Tolerance (1-R^2^) of close to zero would be a concern for multi-collinearity.

A wide range of predictor variables were considered in this study including woman's educational level (no education, primary, and secondary or higher education), working status (whether the woman was working at the time of data collection for remuneration), age (years), marital status (never in union/married, currently married, formerly married), parity (children ever born), wealth index (poor, middle, rich), religion (Orthodox Christian, Muslim, Catholic, Protestant, and Traditional), ethnicity (Tigraway, Oromo, Amhara, Guragie, Somalie, Afar, etc.), history of abortion, woman’s decision-making autonomy, and exposure to media. Exposure to media was categorized as *adequate* if the woman reads newspaper/magazine or listens radio or watches television at least once a week; *inadequate* if the woman reads newspaper/magazine, listens radio or watches television less than once a week. In the 2011 EDHS questions were asked on women’s participation in specific household decisions including on spending respondent’s earnings, household purchases, visits to family and respondent’s healthcare. The decision-making autonomy of women at household level was also considered among the independent variables including decision on own healthcare, large household purchases and visits to relatives. However, we couldn’t include them in the final model due to large missing or invalid values of up to 15% of the sample size.

Other variables treated related to antenatal care, fertility and contraception include history of abortion, current contraceptive use, and knowledge of any contraceptive method. Type of place of residence was used as a control variable.

## Results

The total number of women who had at least one birth and included for this study was 7,759 (weighted = 7905). About 85% of women were from rural areas, majority of the women (69%) were aged 20-34 years while two-thirds had no education. About 91% of the women were currently married and slightly more than half of the households (51%) had between 4 and 6 persons while 37% of the women were of parity 3-5. Regarding ethnicity, Oromo women were the majority (35%); and Orthodox Christians constitute more than 42% of the respondents.

### Prevalence of unintended childbirth

**Figure 1:**
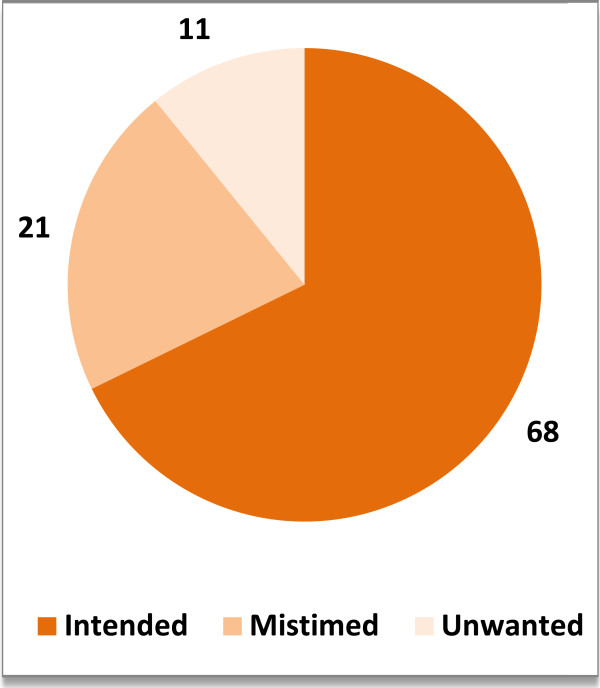
**Prevalence of unintended childbirths in Ethiopia.**

About 32% of the 7905 women had an unintended pregnancy. While 21% were mistimed or were not wanted at the time of conception but later, and 11% were completely unwanted i.e., wanted to stop childbearing (Figure 1).

Table 1 shows the prevalence of unintended childbirth by the socio-demographic characteristics of the respondents who ever had birth and who reported whether their last child was wanted or unwanted.

**Table 1:** **Prevalence of unintended childbirth by socio-demographic characteristics of women 15-49 years, Ethiopia-2011**

Characteristics	Wanted birth	Unwanted birth	Total	N (weighted)
**Age**	*P < 0.001*			
15-19	66.3	33.7	100.0	401
20-34	69.4	30.6	100.0	5480
35-49	63.5	36.5	100.0	2024
**Educational level**	*P = 0.03*			
No education	68.5	31.5	100.0	5268
Primary education	65.6	34.4	100.0	2270
Secondary education	69.6	30.4	100.0	368
**Type of place of residence**	*P = 0.305*			
Urban	66.5	33.5	100.0	1187
Rural	68.0	32.0	100.0	6717
**Respondent currently working**	*P < 0.001*			
No	70.3	29.7	100.0	5131
Yes	63.0	37.0	100.0	2768
**Wealth index**	*P < 0.001*			
Poor	70.7	29.3	100.0	3433
Middle	66.4	33.6	100.0	1628
Rich	64.9	35.1	100.0	2844
**Parity**	*P < 0.001*			
0-2	72.3	27.7	100.0	2751
3-5	66.9	33.1	100.0	2924
6 and above	63.3	36.7	100.0	2200
**Household size**	*P < 0.001*			
1-3	76.3	23.7	100.0	1080
4-6	69.7	30.3	100.0	4056
7 and above	61.6	38.4	100.0	2770
**Religion**	*P < 0.001*			
Orthodox	64.8	35.2	100.0	3327
Protestant	68.3	31.7	100.0	1763
Muslim	71.0	29.0	100.0	2563
Catholic/Traditional/others	69.6	30.4	100.0	247
**Marital status**	*P < 0.001*			
Never in union	13.9	86.1	100.0	72
Married/cohabiting	68.7	31.3	100.0	7185
Formerly married	62.7	37.3	100.0	649
**Ethnicity**	*P < 0.001*			
Afar	95.2	4.8	100.0	63
Amhara	65.4	34.6	100.0	2256
Oromo	61.6	38.4	100.0	2765
Somalie	95.9	4.1	100.0	193
Tigrie	80.5	19.5	100.0	524
Others	72.1	27.9	100.0	2060
**Knowledge of contraceptive method**	*P < 0.001*			
Knows no method	86.0	14.0	100.0	420
Knows at least one method	67.3	32.7	100.0	7339
**Contraceptive use**	*P < 0.001*			
Yes	61.5	38.5	100.0	1810
No	70.1	29.9	100.0	5949
**Media exposure**	*P = 0.001*			
Inadequate	69.1	30.9	100.0	5062
Adequate	65.5	34.5	100.0	2680
**History of abortion**	*P = 0.024*			
Yes	67.3	32.7	100.0	6953
No	71.2	28.8	100.0	803
**Total**	**67.8**	**32.2**	**100.0**	**7905**

Women aged 35-49 years had the highest prevalence of unintended childbirth (37%); while it was least among women aged 15–19 at 34% (P < 0.001). About 86% of never-married women reported having had unintended pregnancy compared to 31% and 37% of currently married/cohabiting and formerly married women, respectively (P < 0.001). Women with parity of two or less had the lowest prevalence of unintended births (28%) compared to 37% among those of parity six and above. Women with household (HH) size of seven and above persons experienced the highest prevalence of unintended births at 38% (P < 0.001).

Women with primary education had higher prevalence of unintended pregnancy (more than 34%) followed by those uneducated (32%) (P < 0.001). The prevalence of unintended birth was higher among women of the richer households (wealth index) at 37% followed by the middle wealth quintiles (34%). There was no statistically significant variation in the prevalence of unintended birth with regards to the woman’s type place of residence.

Ethnically, Oromo and Amhara women had the highest prevalence of unintended pregnancy at more than 38% and 35% respectively. Muslim women had the lowest unintended childbirth (29%) compared to Orthodox Christians (35%) and Protestants (32%) (P < 0.001).

The researchers analysed the association between history of abortion, and knowledge and use of contraception and unintended childbirth. The prevalence of unintended child birth was more than double among those women who know at least one method of contraceptive (32.7%) compared to those who know no method (14.0%) (P = 0.000). Similarly, unintended childbirth was higher among those women who use contraceptives (38.5%) (P = 0.000); and those who had adequate exposure to media (34.5%) (P = 0.001). The prevalence of unintended childbirth was lower among women with no history of abortion (26.1%) compared to those with history of abortion (23.2%) (P = 0.074).

**Figure 2:**
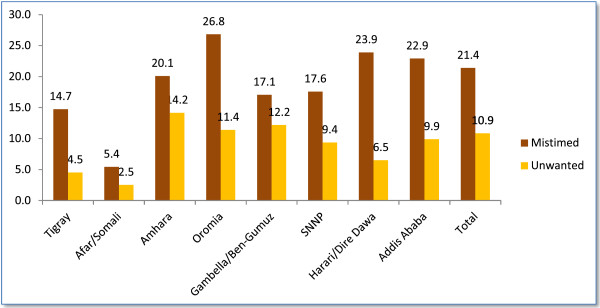
**Proportion of unintended births by region of residence, Ethiopia, 2011.**

With regards to region of residence (Figure 2), the highest unintended birth rates in 2011 were found in Oromiya Region (38% aged 15–49), Harari (35%), Amhara (34%), and Gambella and Addis Ababa each at 33%. The lowest unintended pregnancy rates were found in Somali (8%) and Afar (9%) regions (P < 0.001).

### Socio-economic and demographic determinants of unintended childbirth

In this paper, multivariate logistic regression was run to see the net effects of independent variables over the dependent one. The results of the analysis of the determinants of unintended childbirth are presented in three models (Table 2). Model-I fitted the outcome variable and the socioeconomic variables for urban areas, Model-II for rural and Model-III for both urban and rural combined. Only the geographic, socioeconomic, demographic and other variables, except type of place of residence, which showed statistically significant association in the unadjusted regression models or chi-square tests, were included in the final models. The place of residence did not show any significant association even in the bivariate (chi-square test) analysis (Table 1) but it was included in the final model as a control variable.

**Table 2:** **Binary logistic regression (odds ratio) of unwanted childbirth among women 15-49 years, Ethiopia-2011**

Variables	Model-I	Model-II	Model-III
	AOR [95.0% C.I.]	AOR [95.0% C.I.]	AOR [95.0% C.I.]
**Age** *(RC = 20-34)*			
15-19	1.53 [0.72, 3.25]	1.68 [1.25, 2.25]**	1.66 [1.27, 2.17]****
35-49	1.03 [0.70, 1.53]	0.95 [0.79, 1.14]	0.95 [0.80, 1.12]
**Woman's educational level** *(RC = No education)*			
Primary education	0.97 [0.70, 1.35]	1.17 [1.00, 1.37]	1.13 [0.99, 1.31]
Secondary education	0.50 [0.33, 0.76]**	1.19 [0.69, 2.04]	0.63 [0.47, 0.84]**
**Type of place of residence** *(RC = Urban)*			
Rural			1.10[0.91, 1.33]
**Currently working** *(RC = No)*			
Yes	1.08 [0.82, 1.41]	1.29 [1.12, 1.49]****	1.23 [1.09, 1.40]**
**Wealth index** *(RC = Middle)*			
Poor	3.61 [0.81, 16.04]	1.15 [0.98, 1.36]	1.17 [1.00, 1.38]
Rich	3.11 [1.32, 7.32]*	1.24 [1.05, 1.46]*	1.29 [1.10, 1.51]**
**Parity** *(RC = 0-2)*			
3-5	1.54 [1.09, 2.18]*	1.21 [1.00, 1.46]*	1.29 [1.09, 1.52]**
6 and above	2.56 [1.43, 4.57]**	1.43 [1.13, 1.83]**	1.56 [1.25, 1.94]****
**Household size** *(RC = 1-3)*			
4-6	0.99 [0.70, 1.40]	1.48 [1.15, 1.89]**	1.27 [1.04, 1.55]*
7 and above	1.17 [0.73, 1.86]	2.06 [1.57, 2.70]****	1.74 [1.39, 2.18]****
**Religion** *(RC = Orthodox)*			
Protestant	0.99 [0.63, 1.55]	0.73 [0.58, 0.93]**	0.76 [0.62, 0.93]**
Muslim	0.75 [0.54, 1.05]	0.73 [0.60, 0.89]**	0.77 [0.65, 0.91] **
Catholic/Traditional/others	1.31 [0.37, 4.64]	0.86 [0.59, 1.27]	0.90 [0.63, 1.29]
**Marital status** *(RC = Never in union)*			
Married/cohabiting	0.07 [0.03, 0.18]**	0.18 [0.08, 0.37]**	0.12 [0.07, 0.22]***
Formerly married	0.17 [0.06, 0.45]	0.25 [0.12, 0.53]***	0.20 [0.11, 0.36]***
**Ethnicity** *(RC = Oromo)*			
Afar	0.83 [0.58, 1.18]	0.75 [0.60, 0.93]**	0.76 [0.63, 0.91]**
Amhara	0.11 [0.03, 0.50]**	0.15 [0.10, 0.23]****	0.15 [0.10, 0.23]****
Somali	0.19 [0.09, 0.40]****	0.11 [0.07, 0.18]****	0.13 [0.09, 0.19]****
Tigrie	0.45 [0.26, 0.79]**	0.34 [0.26, 0.46]****	0.37 [0.29, 0.47]****
Others	0.90 [0.60, 1.29]	0.57 [0.47, 0.68]****	0.62 [0.53, 0.73]****
**Knowledge of contraceptive method** *(RC = Knows no method)*			
Knows at least one method	1.04 [0.36, 3.04]	1.78 [1.21, 2.62]**	1.82 [1.25, 2.65]**
**Contraceptive use** *(RC = Yes)*			
No	0.60 [0.45, 0.80]**	0.66 [0.57, 0.78]****	0.64[0.56, 0.73]****
**Media exposure** *(RC = Inadequate)*			
Adequate	1.16 [0.81, 1.65]	0.99 [0.85, 1.15]	1.01 [0.88, 1.16]
**Decision-making on own healthcare** *(RC = Respondent alone)*			
Joint decision	0.81 [0.59, 1.12]	0.72 [0.59, 0.87]**	0.74 [0.62, 0.88]****
Husband or someone else	0.75 [0.48, 1.18]	0.81 [0.66, 1.00]	0.83 [0.68, 1.00]
**History of abortion** *(RC = Yes)*			
No	0.72 [0.46, 1.14]	1.00 [0.81, 1.24]	0.94 [0.78, 1.14]

AOR = adjusted odds ratio, RC = reference category, *p < 0.05, **p < 0.01, ***p < 0.001, ****p < 0.0001.

Model-I shows that urban women with secondary and above education, married/cohabiting and formerly married (divorced or widowed) women, ethnic Afars, Tigraways, Somalies and Amharas, conctraceptive non-users were less likely to experience unintended childbirth. On the contrary, women in the rich wealth quintile, and women with 3-5 and 6+ children ever born were more likely to have unintended event. The rural model (Model-II) shows that younger women, the rich, those currently working, women with parity of 6+, those with household size of 7+ were more likely to have unintended childbirth; while married or cohabiting women, the Amharas, Somalis and Tigraways, and non-contraceptive users were less likely to experience unintended childbirth.

Model-III (Table 2) shows that teenagers (15-19) were nearly two times more likely to have unintended childbirth compared to those aged 20-34 years. Women with secondary education were 37% less likely to have unintended births compared to those with no education; while those in the rich wealth quintile households were 1.3 times more likely to have unintended childbirth. Married or cohabiting women and formerly married women had lower odds of having unintended child birth by about 88% and 80% compared to the never-in-union women. While considering parity, women of 3-5 and 6 and above parity were 1.3 and 1.6 times more likely to experience unintended childbirth compared to the 0-2 parity counterparts. The likelihood of experiencing unintended childbirth was low among Protestants and Muslims with each being 24% and 23% respectively less likely than Orthodox Christians. With regards to ethnic affiliation, Afars, Amharas, Somalis and Tigraways were 34%, 85%, 77% and 63% less likely to experience unintended child birth compared to the Oromos, the largest ethnic group in Ethiopia.

In this model, women’s knowledge of any contraceptive method and history of contraceptive use showed significant association with unintended childbirth. Hence, women who know at least one type of contraceptive method and current users of contraceptives were more likely to have unintended childbirth. History of abortion did not maintain its effect on the final model.

## Discussion

Individuals and couples in Ethiopia want to plan the timing and spacing of their childbearing and to avoid unwanted pregnancies or childbirths for social and economic reasons [11]. Our study shows that in 2011 almost one in three childbirths was unintended of which nearly two-third was mistimed with a similar pattern from other countries including Kenya and Tanzania [14, 16, 17]. At least 27% of last childbirths in every eight out of 11 regional states and city administrations in Ethiopia were unintended. The national unintended childbirth rate is comparable to other African countries. It is less than the rates for Uganda (43%) [5] and neighbouring Kenya (44%); but greater than the rates in Tanzania (21%) and almost the same as that of Zimbabwe (33%).

Unintended childbirth rates were highest among women of rich wealth quintile, aged 35–49, never-in-union/married, with 6+ parity, 7+ household size, and with primary education. These findings both conflict with and support previous study results. Some indicate that unintended child birth is higher among teenage mothers and never-married women [17–19], poor wealth quintile and uneducated women [6, 18, 20–22]; while others support that women in higher wealth quintile [19] and older women [22, 23] had higher odds of unintended childbirths or pregnancies. However, women with secondary and above education were less likely to experience unintended childbirth. This is consistent with results from Bangladesh, Morocco, Colombia and Peru where education showed inverse relation with unintended childbirth [18, 19].

Unlike many previous findings whereby wealth quintile and educational status associate with unintended childbirth in the same direction [6, 18, 20–22], our finding shows a contradicting result in that women of higher wealth quintile households experienced more unintended childbirths while this was true for less educated women. It is worth mentioning here that most of the variables used in the construction of the wealth index in EDHS are characteristics of the urban residents than rural ones. Our analysis shows that 87.5% of the study population in the highest wealth quintiles were urban residents; and unintended childbirths were more prevalent among urban residents than rural ones. Besides, in EDHS educational status was not used in the construction of wealth index. Consequently, the wealth index in EDHS might not be a good proxy reflection of the socioeconomic status of households at national level. Hence, the relationship between wealth quintile and unintended childbirth needs careful interpretation in relation to research findings from different setups or countries.

In this study, place of residence did not strongly associate with unintended childbirth though urban women reported more unintended last births which is in line with research results from India and Bangladesh [21, 23]. The high likelihood of unintended pregnancies among urban women however is contrary to the fact that they have relatively more decision-making power on contraceptive use [24] and higher contraceptive coverage [11] than their rural counterparts. This is also contrary to other studies on the correlates of unintended pregnancy or childbirth [6].

Similar to previous studies from Uganda and Tanzania [6, 18], a study from Eastern Ethiopia indicates that young and unmarried or formerly married women were at higher risk of experiencing unintended pregnancy [15]. The Ethiopia DHS reports and other institutional and community-based research indicate that despite the encouraging or high level of awareness of modern contraceptives [24–27] young people have limited access to quality sexual and reproductive health or family planning services [11, 25, 28]. Another institution-based study indicates that the level of awareness of sexual and reproductive health rights is very low among University students in Ethiopia [29]. In 2011, only 5% of young women aged 15-19 used modern contraceptive methods, the lowest among all the reproductive age groups [11]. Similar to another study to a specific locality in Ethiopia, this study found that high parity is significantly associated with higher prevalence of unintended childbirth.

Interestingly too, ethnicity had statistically significant effect on unintended childbirth. In Ethiopia, being Afar, Amhara, Somali, or Tigraway was associated with a lower likelihood of experiencing unintended childbirth compared to being an Oromo. Oromos constitute the biggest ethnic group in Ethiopia. The Oromos as a whole have continued to experience modest declines in fertility in the recent past, but it has been increasing for Afars and Somalis. Somali and Afar have the lowest proportion of women and men with desire to limit childbearing and have the highest total wanted fertility rate in Ethiopia compared to the Oromos. Somali and Afar regions have the poorest exposure to family planning related media and have the lowest contraceptive utilization rate in Ethiopia at 4% and 9% respectively, versus 25% for Oromiya region. The later has also the highest percentage of unmet need for family planning (30%) [11, 28, 30].

In the current study, Muslim women had lower odds of having unintended childbirth compared to Orthodox Christians. This might be attributed to the tendency of Muslims to have higher fertility compared Christians. Evidence shows that Muslims have the highest fertility in the regions of Ethiopia with equal Muslim and Christian populations (Oromia and Benishangul-Gumuz), but the lowest among Muslim minority regions [31]. Another EDHS data in-depth analysis shows that Muslim women show better decision making power on their own health care as compared to other religious groups [32].

In interpreting this study's findings, it is advisable to consider some of the limitations of the study. The cross-sectional nature of the data does not allow for causal inferences about the relationship between unintended pregnancies or childbirth, and the socioeconomic and demographic correlates. It is important to keep in mind that the analyzed data includes only last births; but possible repeated unwanted pregnancies, those which might have ended in miscarriage, abortion and in some cases turned out to be wanted due to change of mind of the mother or the couple are not measured in this study. As indicated elsewhere, women are more likely to report a current pregnancy rather than a birth that occurred in the past as unintended or unwanted due to difficulty in recall [13]. Women who might have died of the unwanted pregnancy are not also part of the data set. Hence, the current analyses might have underestimated the level of unintended childbirth in Ethiopia. Besides, the categories of mistimed and unwanted births were combined as unintended though they have different concepts. This is common practice in the literature [16, 18].

## Conclusion

The study found a relatively high prevalence of unintended pregnancy or childbirth in Ethiopia. The burden of unintended childbirth in Ethiopia falls more heavily on young, unmarried, multipara, and ethnic majority women and those with primary or no education. Knowledge on any method of family planning and use of contraceptives is also an important factor that is directly related to unintended birth. Exposure to media and wealth status didn’t show any significant net effect on unintended childbirth.

The study suggests an association between socioeconomic and demographic factors and the risk of unintended childbirth. The high proportion of mistimed births in this study is a reflection of the need for spacing, and the relatively lower proportion of unintended births reflects the need for reducing ideal family size and limiting fertility in Ethiopia. A recent study conducted in north-west Ethiopia showed that the majority of contraceptive users prefer child spacing to limiting [27], which supports findings from other parts of Africa [33]. The findings are crucial and imply the need for programs and strategies to strengthen and improve access to contraceptive services, raise educational levels, and related information and communication to bring attitudinal change among the affected women and lower the desire for additional children. Special emphasis should be given to uneducated, unmarried, younger, and multipara women.

Further quantitative and qualitative research on the consequences of unintended (mistimed and unwanted) pregnancy and child birth related to prenatal and perinatal outcomes including abortion, physical and mental health are vital to document process of change in the problem overtime. It would also be advisable to include questions in DHS regarding repeated unwanted pregnancies, outcomes of unwanted pregnancy to measure miscarriage, abortion, live births, neonatal mortalities, its consequences on the physical and psychosocial health of the woman and others. Moreover, qualitative research is needed to understand the underlying reasons for the continuity of the above factors.
